# Supplementary material for: Causal interplay between lactose intolerance and gut microbiota: a combined bidirectional Mendelian randomization and in vivo validation study
Source: Front Nutr. 2026 Jun 1;13:1803337. doi: 10.3389/fnut.2026.1803337 (PMC13265576; doi:10.3389/fnut.2026.1803337)
Supplement: Supplementary file 1 [file Data_Sheet_1.zip › supplementary materials/Forward/leave one out cross validation/ebi-a-GCST90027515.finngen_R12_E4_LACTONAS.pdf]

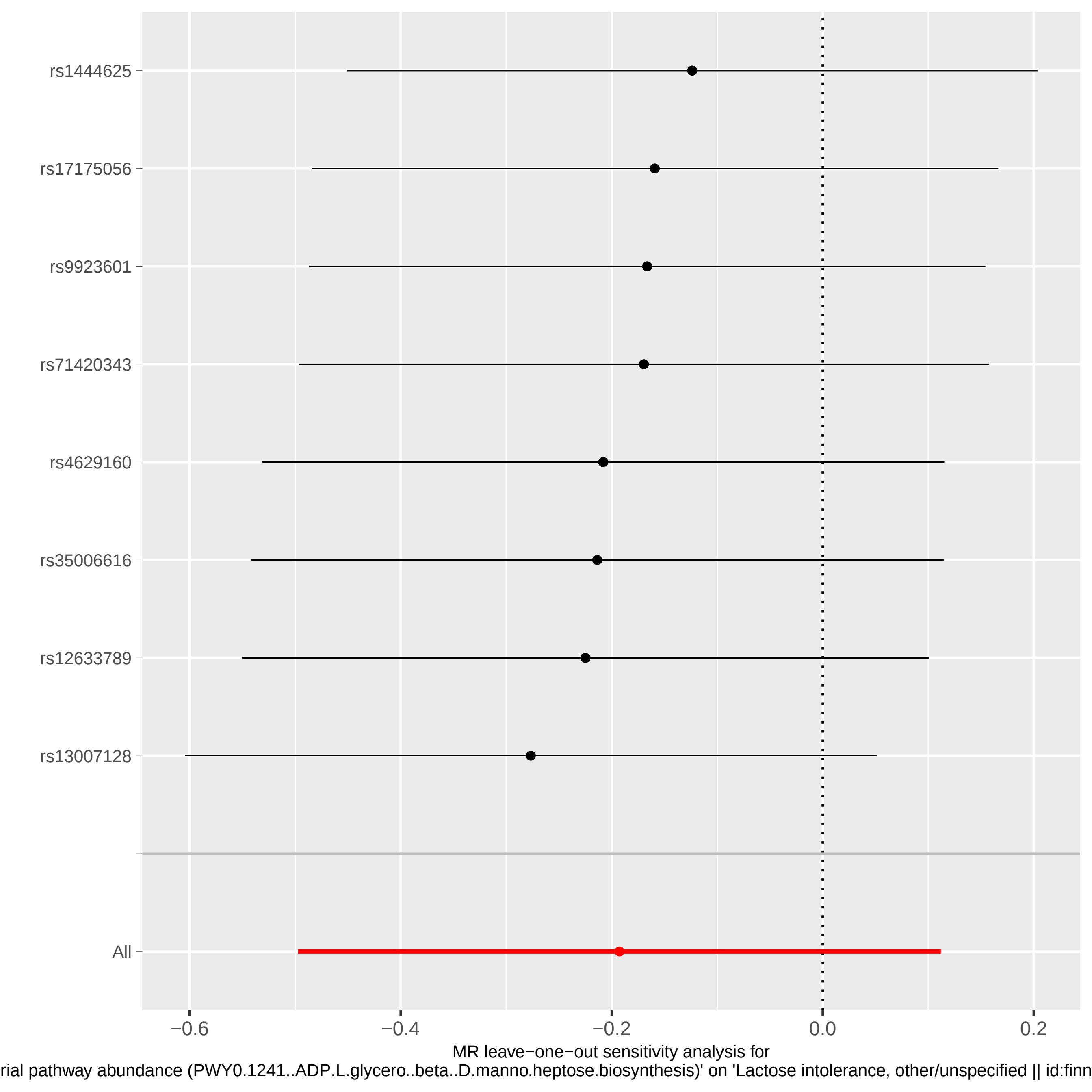
rs1444625
rs17175056
rs9923601
rs71420343
rs4629160
rs35006616
rs12633789
rs13007128
All
−0.6
−0.4
−0.2
0.0
0.2
MR leave−one−out sensitivity analysis for
rial pathway abundance (PWY0.1241..ADP.L.glycero..beta..D.manno.heptose.biosynthesis)' on 'Lactose intolerance, other/unspecified || id:finn
